# A Higher Proportion of Eicosapentaenoic Acid (EPA) When Combined with Docosahexaenoic Acid (DHA) in Omega-3 Dietary Supplements Provides Higher Antioxidant Effects in Human Retinal Cells

**DOI:** 10.3390/antiox9090828

**Published:** 2020-09-04

**Authors:** Manuel Saenz de Viteri, María Hernandez, Valentina Bilbao-Malavé, Patricia Fernandez-Robredo, Jorge González-Zamora, Laura Garcia-Garcia, Nahia Ispizua, Sergio Recalde, Alfredo Garcia-Layana

**Affiliations:** 1 Department of Ophthalmology, Clinica Universidad de Navarra, 31008 Pamplona, Spain; msaenzdevit@unav.es (M.S.d.V.); vbilbao@unav.es (V.B.-M.); jgzamora@unav.es (J.G.-Z.); aglayana@unav.es (A.G.-L.); 2 Retinal Pathologies and New Therapies Group, Experimental Ophthalmology Laboratory, Department of Ophthalmology, Clinica Universidad de Navarra, 31008 Pamplona, Spain; mahersan@unav.es (M.H.); mgarcia.6@alumni.unav.es (L.G.-G.); nispizua@alumni.unav.es (N.I.); srecalde@unav.es (S.R.); 3 Navarra Institute for Health Research, IdiSNA, 31008 Pamplona, Spain; 4 Red Temática de Investigación Cooperativa Sanitaria en Enfermedades Oculares (Oftared), 31008 Pamplona, Spain

**Keywords:** eicosapentaenoic acid (EPA), docosahexaenoic acid (DHA), oxidative stress, diabetic retinopathy, retinal pigment epithelium

## Abstract

Retinal pigment epithelium (RPE) is a key regulator of retinal function and is directly related to the transport, delivery, and metabolism of long-chain n-3 polyunsaturated fatty acids (n3-PUFA), in the retina. Due to their functions and location, RPE cells are constantly exposed to oxidative stress. Eicosapentaenoic acid (EPA) and docosahexaenoic acid (DHA) have shown to have antioxidant effects by different mechanisms. For this reason, we designed an in vitro study to compare 10 formulations of DHA and EPA supplements from different origins and combined in different proportions, evaluating their effect on cell viability, cell proliferation, reactive oxygen species production, and cell migration using ARPE-19 cells. Furthermore, we assessed their ability to rescue RPE cells from the oxidative conditions seen in diabetic retinopathy. Our results showed that the different formulations of n3-PUFAs have a beneficial effect on cell viability and proliferation and are able to restore oxidative induced RPE damage. We observed that the n3-PUFA provided different results alone or combined in the same supplement. When combined, the best results were obtained in formulations that included a higher proportion of EPA than DHA. Moreover, n3-PUFA in the form of ethyl-esters had a worse performance when compared with triglycerides or phospholipid based formulations.

## 1. Introduction

Diabetes mellitus (DM) epidemic is a global public health problem and the leading cause of preventable blindness in the working-age population [1]. The International Diabetes Federation, in 2019, stated that DM affected an estimated 425 million adults worldwide and this number is likely to increase over the next few years due to urbanization, increased obesity prevalence, and sedentary lifestyles. According to epidemiologic predictions 1 in 10 adults across the globe, will live with diabetes by 2045 [2]. The physiopathology of DR is complex and several interconnecting biochemical pathways have been proposed as contributors in its development. These include the polyol pathway [3], non-enzymatic glycation [4], activation of protein kinase C (PKC) [5], oxidative stress [6,7,8,9,10], and inflammation through proinflammatory cytokines, chemokines, and other inflammatory mediators [11,12]. Among these, oxidative stress and inflammation are major causal factors involved in the endothelial dysfunction of the retina microvasculature that occurs in DR [6,7,8,9,10]. In addition, this chronic low-grade inflammation could finally lead to neovascularization [13]. While improvements in treatment have reduced the macro and microvascular complications of the disease, the increasing number of diabetic patients combined with the extended life expectancy means that more patients will live long enough to develop DR. In fact, it is expected that the number of people with DR will grow from 126.6 million in 2010 to 191.0 million by 2030 [14].

Metabolic abnormalities of diabetes cause mitochondrial superoxide overproduction [15]. This is the central and major mediator of diabetes endothelial dysfunction and tissue damage, with several pathways involved in the pathogenesis: Polyol pathway, increased formation of advanced glycation end-products (AGEs), increased expression of the receptor for AGEs and its activating ligands, activation of protein kinase C (PKC) isoforms, and overactivity of the hexosamine pathway [15].

According to the above, long-chain n-3 polyunsaturated fatty acids (n3-PUFA), including eicosapentaenoic acid (EPA), and docosahexaenoic acid (DHA) have been studied as an alternative therapy for retinal diseases due to their pleiotropic effects including anti-inflammatory, antioxidant, antiproliferative, and antiangiogenic properties. They are essential fatty acids in the human diet that exert anti-inflammatory and antioxidant effects by binding cell membrane receptors to affect downstream mediators and alter gene expression [16]. DHA is a major structural lipid in the sensory and vascular retina. In fact, the highest body concentrations of DHA per unit weight are found in phospholipids of retinal photoreceptor outer segments. Retinal pigmented epithelium (RPE) is a polarized epithelial monolayer known to synthesize DHA [17]. Moreover, the RPE plays an important role in regulation and delivery of DHA from the plasma to the photoreceptors [18]. EPA is converted intracellularly to DHA at low basal levels, yet exogenous EPA supplementation does not increase DHA levels in human plasma [19]. Rather than rapidly converting to DHA, EPA seems to have a clinically relevant biological activity itself, distinct from that of DHA [20,21,22].

Several clinical trials have demonstrated the beneficial effects of the administration of n3-PUFA in the development of DR. J. Howard-Williams et al. found that poorly controlled patients with low levels of n3-PUFA intake had a significantly greater frequency of retinopathy [23]. Similarly, a reduced severity of DR in well-controlled diabetes patients was observed with increasing n3-PUFA intake [24]. The primary prevention of cardiovascular disease with a Mediterranean diet (PREDIMED) demonstrated that participants taking at least 500 mg/day of long-chain v-3 PUFAs, showed a 48% relatively reduced risk of incident sight-threatening DR compared with those not fulfilling this recommendation [25]. Moreover, the addition of DHA supplement to intravitreal ranibizumab was effective to achieve better sustained improvement of central subfield macular thickness compared with ranibizumab alone [26].

In diabetic animal models, the ability of n3-PUFAs, especially EPA and DHA, to suppress IL-6, TNF-a, ICAM-1, MCP-1, and VEGF production has been demonstrated, as well as the reduction of free radical generation and the restoration of antioxidant homeostasis [27,28,29,30]. According to the conclusions of a study conducted by Mahmoudabadi and Rahbar [31], the administration of EPA increases several endogenous antioxidant enzymes, namely superoxide dismutase and glutathione peroxidase, while simultaneously decreasing the levels of malondialdehyde, a classical biomarker of oxidative stress, in type II diabetic patients.

Despite their similarities in their nutritional sources and most of their biological actions, the type of n3-PUFA formulation seems to matter in treating different diseases. DHA is associated with decreased Alzheimer disease risk in humans [32]. Conversely, EPA has a more therapeutic effect in treating depression [19,20,21] while cardiovascular outcomes have been shown to improve after the intake of combined EPA and DHA supplements [33]. Nevertheless, it should be noted that many of the observational studies published in DR only supplement one type of n3-PUFA (EPA or DHA), while some of them did not measure the type of omega 3 consumed, but rather frequency of fish oil consumption. In addition, the efficacy of the use of different formulations of DHA and EPA have not been studied to date. Currently, the primary dietary source of these fatty acids is fish oil; however, since the global consumption cannot be satisfied due to the increasing demand, alternative sources such as microalgae have emerged [34,35].

Given this lack of evidence, we designed an in vitro study to compare 10 formulations of DHA and EPA supplements from different origins, and assess their safety profile and their ability to rescue retinal pigment epithelium (RPE) cells from the oxidative and inflammatory conditions seen in the DR.

## 2. Materials and Methods

### 2.1. Cell Culture

ARPE-19, obtained from the American Type Culture Collection (CRL-2302, ATCC^®^, Manassas, VA, USA), were grown to confluence in a standard incubator at 37 °C in humidified 5% CO_2_ condition in a DMEM/F12 medium (1:1) (Sigma-Aldrich, St. Louis, MO, USA) containing 10% fetal bovine serum (FBS; Sigma-Aldrich, St. Louis, MO, USA), 1% fungizone, and L-glutamine penicillin-streptomycin (Sigma-Aldrich, St. Louis, MO, USA). Cells were passaged every 3–4 days with 0.25% Trypsin-EDTA (Invitrogen, Carlsbad, CA, USA). For all assays, cells were grown to 100% confluence with the exception of BrdU, which requires non-confluent cells grown for 1–2 days.

### 2.2. Omega-3 Supplementation

Fatty acids (FA) were conjugated to albumin to solubilize them and to replicate the in vivo environment. Cells were seeded with a lipid-free bovine serum albumin (BSA) media in a 2:1 FA/BSA ratio.

Ten different omega-3 supplements were tested grouped in three parts as shown in Table 1: I: EPA and DHA, separately; II: EPA and DHA combined in different proportions and in the form of ethyl esters (EE) or triglycerides (TG); III: DHA-TG combined with DHA phospholipids (PL) from different origins (marine or vegetable). All the supplements were provided by Théa Laboratories (Clermont-Ferrand, France). Details of the composition of each formulation and identification of groups are displayed in Table 1.

### 2.3. Immunofluorescence Detection of Zonula Occludens

The effect of all FA groups on the integrity of the epithelial tight junctions was determined by ZO-1 immunofluorescence to determine if any formulations compromised these intercellular unions. One hundred thousand ARPE-19 cells were seeded onto polycarbonate inserts (Corning^®^ Transwell^®^, Phoenix, AR, USA,) and kept in a culture with 1% DMEM for four weeks to allow epithelial polarization. Supplements were then added to ARPE-19 cells, which were fixed in cold methanol after 24 h. After three washes with 1% PBS, cells were submerged in 1% PBS-BSA for 20 min to block non-specific bonds before incubation with the polyclonal ZO-1 anti-rabbit antibody (1:100; Life Technologies, Gaithersburg, MD, USA) in 1% PBS-BSA at 4 °C for 24 h. The cells were washed with 1% PBS (three times of 5 min) and incubated with the secondary antibody goat anti-rabbit Alexa Fluor^®^ 488 (1:250; A11008 Invitrogen, Thermo Fisher, Madrid, Spain) in 1% PBS-BSA for 1 h in the dark at room temperature. TOPRO-3 was used for nuclear staining. Membranes were cut from the transwell insert with a scalpel and placed on a microscope slide and mounted with PBS-Glycerol 1:1. Images were captured in the Z-stack mode with a laser scanning confocal imaging system (Zeiss LSM-510 Meta, Oberkochen, Germany) using a 40× objective.

### 2.4. Cellular Viability and Proliferation Assays

The 3-(4,5-dimethylthiazol-2-yl)-2,5-diphenyltetrazolium bromide (MTT) reduction assay was used to determine cellular viability. Experiments were carried out on 96-well plates seeded with 10,000 ARPE-19 cells per well. Once cells were confluent, a culture medium was changed to 1% FBS and maintained for two days. At this point, cells were exposed to 100 µM of each supplement. After 96 h, cell viability was analyzed using the CellTiter 96^®^ AQueous One Solution Cell Proliferation Assay (Promega, Madison, WI, USA), following the manufacturer’s instructions. At 77 h MTT absorbance was determined at 450 and 540 nm with a Sunrise-basic Microplate reader (Tecan, Austria).

To assess the effect of the different formulations under oxidative stress conditions, at 72 h some cells were additionally exposed to 800 uM H_2_O_2_.

Cell proliferation was quantified by BrdU incorporation into the ARPE-19 genome using the Calbiochem^®^ BrdU Cell Proliferation Assay (Calbiochem, La Jolla, CA, USA). Between 10,000 to 20,000 cells were seeded onto 96-well plates. After 24 h, 50 µM of the 10 supplements were added to the cells. BrdU was performed 48 h later, according to the manufacturer’s protocol. Some cells were also challenged with H_2_O_2_ (as described above).

### 2.5. Reactive Oxygen Species (ROS) Detection

To measure the production of reactive oxygen species (ROS) generated by the n3-PUFA, the DCF (2′,7′-dichlorofluorescein) (H2DCFDA) test was used. Upon being oxidized, DCF emits fluorescent light at a wavelength of 540 nm when excited at 480 nm. The emitted fluorescence is captured and quantified by fluorometry. Once ARPE-19 cells reached confluence, a 10% FBS culture medium was replaced by 1% FBS medium for 24 h. Next, cells were exposed to the different supplements during 24 h. Apigenine was used as a positive control. Following this incubation, H2DCFDA was added to the medium for 30 min; cells were then harvested from the wells and transferred into a black 96-well plate to measure the emitted fluorescence.

### 2.6. Caspase-3 Immunofluorescence (IF)

The protein caspase-3 is an essential mediator of the activation of the apoptotic signaling cascade. To detect if the omega-3 formulations activated cell death by apoptosis, this early apoptotic mediator was visualized by confocal immunofluorescence microscopy. For these experiments, 50,000 ARPE-19 cells were cultured on 1 cm diameter cover slips (CB 100RA1, Menzel-Gläser, Braunschweig, Germany) until confluence. Then, cells were treated with a supplement for 24 h and subjected to 800 µM H_2_O_2_ for 3 h to activate the caspase-mediated apoptotic cascade (positive control). At the end of the experiment, glass coverslips were fixed and permeabilized in cold methanol. Afterwards, coverslips were washed with 1% PBS and submerged in 1% PBS-BSA for 20 min to block nonspecific bonds. The polyclonal rabbit anti-caspase-3 (G7481, Promega, WI, USA) was used at a 1:100 dilution in 1% PBS-BSA at 4 °C for 24 h. The coverslips were then washed with 1% PBS and incubated with an Alexa Fluor^®^ 488 goat anti-rabbit IgG antibody (A11008, Invitrogen, Carlsbad, CA, USA) diluted in 1% PBS-BSA in the darkness for 1 h. TOPRO-3 was used as a nuclear marker. Next, the coverslips were mounted on microscope slides with PBS-glycerol and gelatin at 1:1 and observed under the confocal fluorescent microscope (LSM 750, Carl Zeiss, Oberkochen, German).

For caspase-3 quantification, each coverslip was divided into eight sectors and one image at 40× was acquired from alternate sectors. A total of four images were acquired from each cover slip and were analyzed using the LSM Zeiss software to compile them into one merged image, and caspase-3 granules per nucleus were quantified using a home-made plugin tool developed for Fiji/ImageJ, an open-source Java-based image analysis software. The plugin has been developed by the Imaging Platform of the CIMA Universidad de Navarra.

### 2.7. Wound Healing Cell Migration Assay

A wound healing assay was used to quantify ARPE-19 migration in the presence of n3-PUFA under standard conditions. For these experiments, 150,000 cells were seeded onto 24-well culture plates until confluence. A linear wound was then created in the middle of each well using a 20 µL sterile micropipette tip. Culture media was replaced to eliminate floating cells and debri and the different n3-PUFA formulations were added. Five points on each well were captured every hour for a total of 72 h using an automatic phase contrast inverted microscope equipped with a digital camera (Carl Zeiss, Oberkochen, Germany). Every set of images was analyzed using the Fiji software (a distribution of ImageJ) V1.48q (Fiji Wound Healing Tool by Nathalie Cahuzac, and Virginie Georget, http://dev.mri.cnrs.fr/projects/imagej-macros/wiki/Wound_Healing_Tool) to determine the speed of closure.

### 2.8. Western Blotting for Vascular Endothelial Growth Factor (VEGF)/Pigment Epithelium Derived Factor (PEDF) Ratio

Following 24 h of treatment with the different omega-3 formulations, 5 µg of ARPE cells homogenates were mixed with a Laemmli buffer (62.5 mM Tris-HCl, pH 6.8; 2% SDS; 10% glycerol; 0.1% bromophenol blue) and boiled for 5 min. Samples were separated on 12% SDS-PAGE gels and transferred to a nitrocellulose membrane. After blocking with 5% skimmed milk (*w*/*v*), 0.1% Tween-20 (*w*/*v*) in TBS for 1 h at room temperature, membranes were exposed to the primary antibodies (0.2 μg/μL, monoclonal anti-VEGF, sc7269, Santa Cruz Biotechnology Inc., Santa Cruz, CA, USA and 1:1000 monoclonal anti-PEDF; MAB1059; Millipore, Burlington, MA, USA), at room temperature for 1 h. Membranes where then incubated at room temperature for 1 h with a horseradish peroxidase-conjugated goat anti-mouse antibody (sc2005; 0.4 µg/µL, Santa Cruz Biotechnology Inc., Dallas, TX, USA). Signals were detected with an enhanced chemiluminescence (ECL) kit (ECL Western blotting detection kit, GE Healthcare, Fairfield, CT, USA) and with ImageQuant 400 (GE Healthcare). The relative intensities of the immunoreactive bands were analyzed with Quantity One software (version 4.2.2, Bio-Rad Laboratories, Hercules, CA, USA). The loading was verified by Ponceau S red, and the same blot was stripped and reblotted with an anti-β-actin monoclonal antibody (Sigma-Aldrich, St Louis, Mo, USA) to normalize the VEGF and PEDF levels. Protein levels were used to calculate the VEGF/PEDF ratio.

### 2.9. Statistical Analysis

For quantitative variables, and after assessing application conditions, all parameters were subjected to the one-way analysis of variance (ANOVA) followed by the Bonferroni post-hoc test. All groups were normalized by each pass and compared versus a control group. Data are expressed as mean ± SEM. A difference *p* < 0.05 was considered statistically significant. GraphPad Prism 6.0 (GraphPad Prism Software Inc., San Diego, CA, USA) was used for statistical analysis.

## 3. Results

### 3.1. Effect of Omega-3 Supplements on Epithelial Integrity

**Figure 1:**
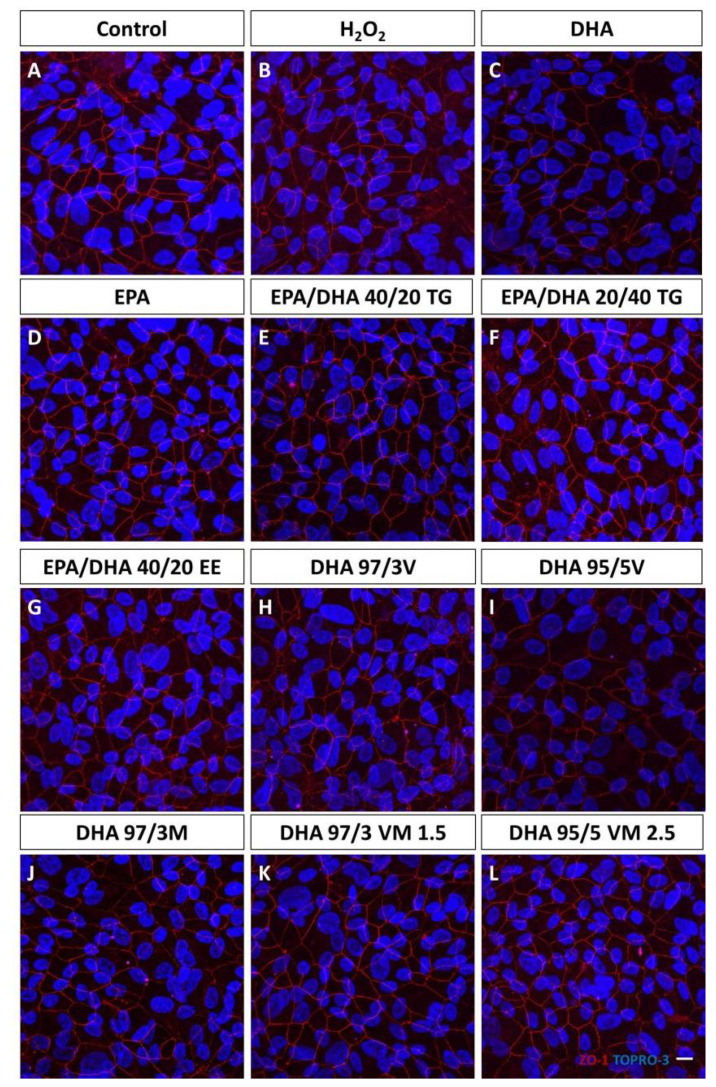
Effect of omega-3 supplements on epithelial tight junctions. ZO-1 (red) immunofluorescence were not affected by any of the omega-3 treatments. Nuclei are stained with TOPRO-3. Scale bar 20 µm. (**A**): Control group; (**B**): H_2_O_2_ treatment group; (**C**): DHA (Docosahexaenoic acid) group; (**D**): EPA (Eicosapentaenoic acid) group; (**E**): EPA/DHA 40/20; TG (Triglycerides) group; (**F**): EPA/DHA 20/40 TG group; (**G**): EPA/DHA 40/20 EE (Ethylesters) group; (**H**): DHA 97/3V (Vegetable) group; (**I**): DHA 95/5V group; (**J**): DHA 97/3M (Marine) group; (**K**): DHA 97/3 VM 1.5 group; (**L**): DHA 95/5 VM 2.5 group.

The distribution of the ZO-1 was similar after treatment with the 10 different n3-PUFA supplements. No difference was found among any group of study (Figure 1).

### 3.2. Effect of Omega-3 Supplements on Viability and Proliferation in ARPE-19

**Figure 2:**
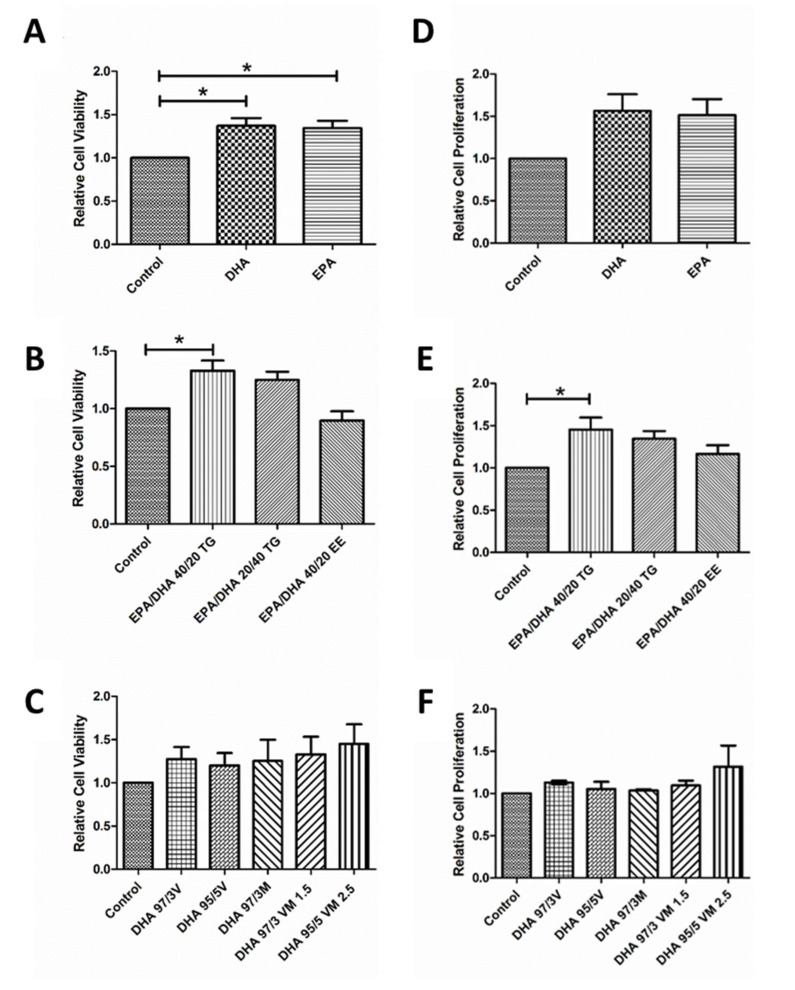
Graphs showing relative cell viability (**A**–**C**) and proliferation (**D**–**F**) compared to a control group under normal conditions of groups 1, 2, and 3 of eicosapentaenoic acid/docosahexaenoic acid (DHA/EPA) formulations, respectively (**D**–**F**). For comparisons, one-way ANOVA with the Bonferroni post-hoc test were used. Data are expressed as mean ± SEM. * *p* < 0.05. EPA: Eicosapentaenoic acid; DHA: Docosahexaenoic acid; TG: Triglycerides; EE: Ethylesters; PL: Phospholipids. V: Vegetable. M: Marine.

First, we wanted to evaluate the effect of Docosahexaenoic acid (DHA) and Eicosapentaenoic acid (EPA) on cell viability and proliferation. Treatment with both n3-PUFA produced a significant increase in viability (Figure 2A) of ARPE-19 cells compared to untreated controls. Proliferation also seemed to increase, but the effect was not statistically significant (Figure 2D). Secondly, we assessed the effect of different combinations of DHA and EPA on these cellular parameters. Interestingly, only the EPA/DHA 40/20 TG combination was able to significantly increase both viability (Figure 2B) and proliferation (Figure 2E), when compared to the untreated cells. Finally, we evaluated five different supplements containing a mixture of DHA in the form of TG and PL (PL from marine, vegetable, or mixed origin). In this last set of experiments, none of the formulations produced a significant change in cell viability (Figure 2C) or proliferation (Figure 2E).

### 3.3. Effect of Omega-3 Supplements on Cell Viability and Cell Proliferation under Oxidative Stress and Inflammatory Conditions

**Figure 3:**
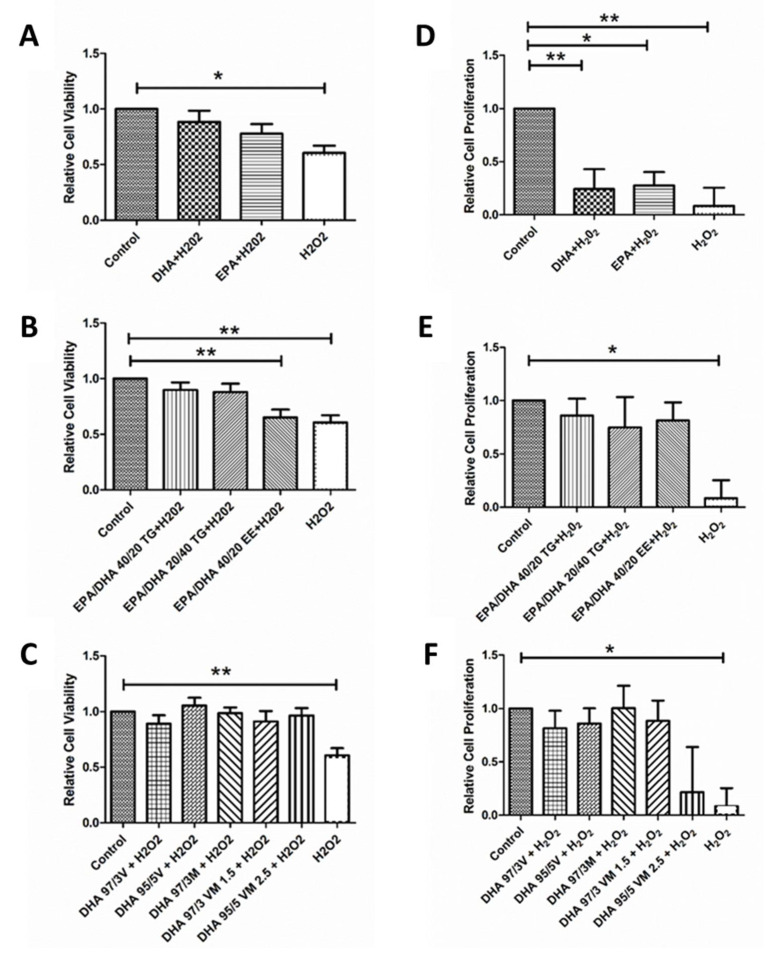
Graphs showing relative cell viability (**A**–**C**) and proliferation (**D**–**F**) results for ARPE-19 cells treated with different DHA and EPA treatments groups under oxidative stress conditions (H_2_O_2_ For comparisons, one-way ANOVA with the Bonferroni post-hoc test were used. Data are expressed as mean ± SEM. * *p* < 0.05 and ** *p* < 0.01. EPA: Eicosapentaenoic acid; DHA: Docosahexaenoic acid; TG: Triglycerides; EE: Ethylesters; PL: Phospholipids. V: Vegetable. M: Marine.

In order to replicate the local oxidative environment of the diabetic retina, we subjected some ARPE-19 cells to H_2_O_2_ and measured the response after n3-PUFA treatment. As expected, exposure to 800 µM H_2_O_2_ produced a significant decrease in cell viability and proliferation. Treatment with EPA and DHA was able counteract the H_2_O_2_ induced decrease on cell viability (Figure 3A), but their effect on cell proliferation, although positive, was not statistically significant (Figure 3D). On the contrary, both n3-PUFA were able to significantly counteract the effect of H_2_O_2_ on cell proliferation (Figure 3D). In the combined formulations, all supplements were able to significantly reverse the oxidative effect of H_2_O_2_ on viability and proliferation, with the exception of the 40/20 EPA/DHA EE supplement that was not able to significantly mitigate the effect of H_2_O_2_ on cell viability (Figure 3B,C,E,F).

### 3.4. Effect of Omega-3 Supplements on ROS Production

**Figure 4:**
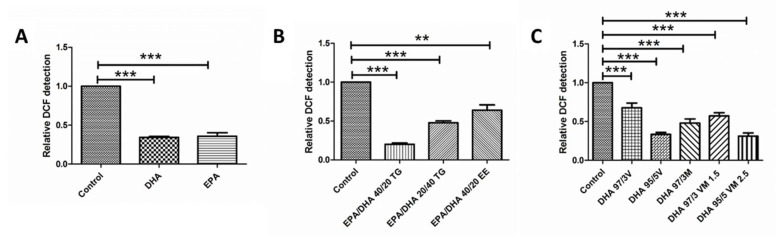
Graphs showing relative Dichloride fluoresceine (DCF) detection results for ARPE-19 cells in groups 1, 2, and 3 of DHA/EPA formulations, respectively (**A–C**). For comparisons, one-way ANOVA with the Bonferroni post-hoc test were used. Data are expressed as mean ± SEM. ** *p* < 0.01 *** *p* < 0.001. EPA: Eicosapentaenoic acid; DHA: Docosahexaenoic acid; TG: Triglycerides; EE: Ethylesters; PL: Phospholipids. V: Vegetable. M: Marine.

Both DHA and EPA, alone or in combined formulations, produced a significant decrease in ROS when compared with the untreated controls. The same effect was observed in cells treated with the different DHA TG+PL formulations. The EPA/DHA 40/20 TG and DHA 95/5 VM 2.5 formulations had a stronger antioxidant effect, but this difference was not statistically significant when compared with the other combinations (Figure 4A–C).

### 3.5. Effect of Omega-3 Supplements on Caspase-3 in ARPE-19

**Figure 5:**
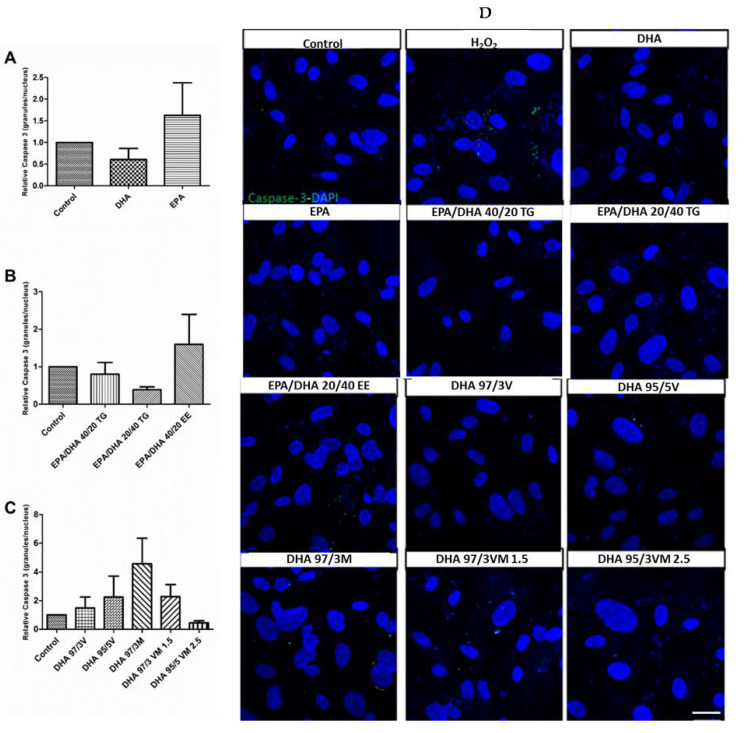
Graphs showing relative caspase-3 expression (positive granules per nucleus) (**A–C**) results for ARPE-19 cells treated with different groups 1, 2, and 3 of DHA/EPA formulations, respectively. For comparisons, one-way ANOVA with the Bonferroni post-hoc test were used. (**D**) Scale bar 20 µm Data are expressed as mean ± SEM. EPA: Eicosapentaenoic acid; DHA: Docosahexaenoic acid; TG: Triglycerides; EE: Ethylesters; PL: Phospholipids. V: Vegetable. M: Marine.

In order to test the safety of the different supplements, we evaluated their capacity to activate the early apoptotic mediator caspase-3. As expected, none of the omega-3 formulations induced a significant change in caspase-3 activation, when compared to the untreated control cells (Figure 5).

### 3.6. Effect of Omega-3 Supplements on Wound Healing Cell Migration Assay

**Figure 6:**
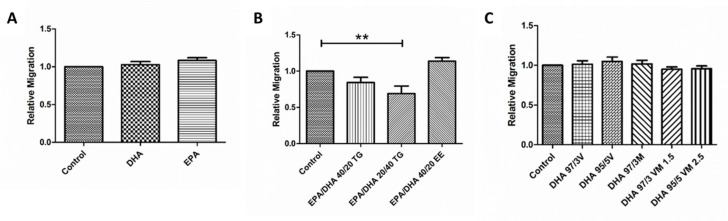
Graphs showing relative migration ratio (**A–C**) results for ARPE-19 cells treated with different groups 1, 2, and 3 of DHA/EPA formulations, respectively. For comparisons, one-way ANOVA with the Bonferroni post-hoc test were used. Data are expressed as mean ± SEM. ** *p* < 0.01. EPA: Eicosapentaenoic acid; DHA: Docosahexaenoic acid; TG: Triglycerides; EE: Ethylesters; PL: Phospholipids. V: Vegetable. M: Marine.

Treatment with EPA or DHA did not produce any significant change in the migration capacity of ARPE-19 cells (Figure 5D). In the EPA/DHA formulations, the EPA/DHA 20/40 TG supplement produced a small, but significant decrease in the speed of wound closure, when compared with untreated cells. The rest of the combined EPA/DHA formulations and those combining DHA in TG and PL forms did not produce any significant changes in this cellular function (Figure 6).

### 3.7. Effect of Omega-3 Supplements on the VEGF/PEDF Ratio

**Figure 7:**
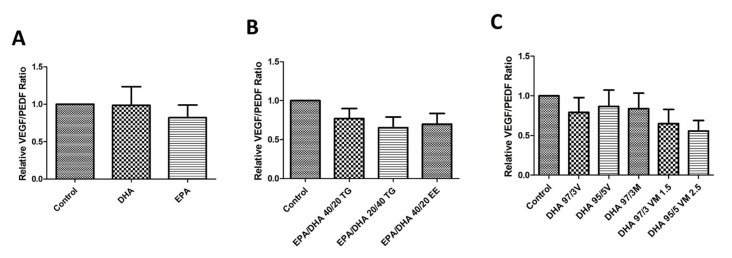
Graphs showing relative VEGF/PEDF ratio (**A–C**) results for ARPE-19 cells treated with different groups 1, 2, and 3 of DHA/EPA formulations, respectively. For comparisons, one-way ANOVA with the Bonferroni post-hoc test were used. Data are expressed as mean ± SEM. EPA: Eicosapentaenoic acid; DHA: Docosahexaenoic acid; TG: Triglycerides; EE: Ethylesters; PL: Phospholipids. V: Vegetable. M: Marine.

Treatment with the different omega-3 formulations did not produce a significant effect in the protein levels of VEGF or PEDF. However, combined formulations (especially those with EPA+DHA), showed a tendency to decrease the VEGF/PEDF ratio when compared with untreated controls, but these differences were not statistically significant (Figure 7).

## 4. Discussion

RPE is a key regulator of retinal function and is directly related to the transport, delivery, and metabolism of n-3 PUFA in the retina. For this reason, we aimed to evaluate the effect of different formulations of DHA and EPA on this important cell type. Both n-3 PUFAs produced favorable effects on RPE cells by increasing cell viability and proliferation, reducing the production of ROS and decreasing oxidative damage induced by H_2_O_2_.

Recent research has demonstrated that the long chain n-3 PUFA, has antiangiogenic, anti-vasoproliferative, and neuroprotective actions on factors and processes implicated in the pathogenesis of degenerative/vascular retinal diseases of greatest public significance, including DR [18]. It has been demonstrated that diets with high levels of n-3 PUFA, have known anti-inflammatory properties [36] due to their ability to promote the gene expression of various inflammatory mediators via different intracellular signaling pathways [37] leading to the inhibition of the expressions of pro-inflammatory cytokines, leukocyte chemotaxis, and adhesion molecules. It is also known that they regulate the production of eicosanoids such as prostaglandins and leukotrienes and increase the synthesis of anti-inflammatory mediators such as resolvins, protectins, and maresins [38]. All these anti-inflamatory mechanisms add to their antioxidant effects by increasing the bioavailability of nitric oxide (NO) and the expression of superoxide dismutase and glutathione peroxidase, known as endogenous antioxidant enzymes, while decreasing the level of biomarkers of oxidative stress, such as malondialdehyde, in type II diabetic patients [30].

Many of the n3 supplements used in the routine clinical practice have a combination of EPA and DHA. For this reason, we explored the effect of supplements that mixed EPA and DHA in different proportions. Interestingly, EPA/DHA 40/20 TG was the only formulation that showed a significant increase in viability and proliferation of ARPE-19 cells and the most favorable antioxidant effect. This observation is in agreement with previous publications that have shown different effects of omega-3 supplements, depending on the proportion of EPA/DHA. In a study conducted on Wistar rats, it was found that the dietary intervention with 1:1 and 2:1 EPA/DHA supplements were the most effective treatments to reduce inflammation and oxidative stress when compared with a 1:2 EPA/DHA formulation [39]. Similar results were reported in a study performed in spontaneously hypertensive obese rats, where EPA/DHA supplementation at the ratios of 1:1 and 2:1 were more effective than a 1:2 formulation, lowering plasma total cholesterol and LDL concentrations, decreasing inflammation, and increasing the activity of antioxidant enzymes [40]. The reasons that underlie these differences are not completely clear, but it has been suggested that the higher unsaturation level of DHA may increase the susceptibility of the molecule to be oxidized compared to EPA, rendering a higher level of free radicals [39]. Furthermore, differences in their influence on transduction pathways, the release of inflammatory cytokines, and the expression of genes involved in lipid metabolism have also been reported [40]. In the field of ophthalmology, one article compared the effect of two different EPA/DHA formulations (1/4.5 and 1.5/1) as adjuvants to topical tacrolimus in a model of keratoconjunctivitis sicca (dry eye disease) in dogs [41]. Authors reported better clinical and biochemical outcomes after supplementation with the oral formulation containing a higher proportion of EPA.

However, the proportion of EPA/DHA is not the only factor that distinguishes one supplement from the other, and omega-3 formulations vary depending on whether they are present as TG, EE, or PL. In the body, long PUFA stores exist mainly as PL and TG and in the retina, the latter represents the predominant lipid class [18]. Although most of the large interventional studies in the field of omega-3 supplements have been conducted with EPA/DHA EE, several authors have commented on their lower bioavailability when compared to TG and PL formulations [42,43,44]. Possible explanations include differences in their digestion and absorption [42]. EPA and DHA may be obtained directly through the diet or can be biosynthesized from linoleic acid (an 18-carbon essential fatty acid) in the liver or the retina. However, the efficiency of tissue accretion is highest when they are ingested in the preformed state [18]. n-3 PUFA are hydrolyzed by pancreatic enzymes and are re-esterified to triglycerides and phospholipids within the intestinal epithelium. These triglycerides and phospholipids are integrated to chylomicrons and very low density lipoproteins (VLDLs) which are transported to the choriocapillaris. Further transport from the choriocapillaris to the RPE and the inner segments of photoreceptors appears to be mediated by high affinity receptors [18]. Unlike TG and PL, which are hydrolyzed mainly by a colipase dependent pancreatic lipase, EE requires additional digestion with carboxyl ester lipase, a step that can slow down their absorption by 10 to 50 times [44,45]. In our experiments, the supplement with EPA/DHA in the form of EE, did not improve cell viability or proliferation and showed a lower antioxidant effect, despite having the same proportion of EPA/DHA (40/20) than the TG supplement with the most favorable outcomes. These differences could be clinically relevant, as suggested by data from The Age-Related Eye Disease Study 2 (AREDS2), where supplementation with a 650 mg EPA/350 mg DHA EE formulation failed to show clinically significant benefits [46].

As recent evidence has suggested that dietary EPA-DHA PL are superior to TG and EE forms in exerting their functional mechanisms [43], we evaluated the effect of DHA formulations that combined TG and PL. The most relevant effect of these formulations was their antioxidant capacity. They decreased the production of ROS production, and increased viability and proliferation of cells challenged with H_2_O_2_. This effect is important as the oxidative stress in the human eye is also primarily due to H_2_O_2_, which is naturally generated in RPE cells by solar radiation and POS phagocytosis [47]. Regarding the origin of DHA PL, some authors have suggested that n3-PUFA from a marine origin might be more beneficial than those from a vegetable origin [39,43,48]. However, in our experiments we did not find an association between the origin of the DHA PL and their results. This is probably because the proportion of PL in our formulations was small (3% or 5%).

The above-mentioned beneficial effects of the n3-PUFA have been demonstrated in several clinical studies. In patients with DR and well-controlled diabetes, increasing the n3-PUFA intake was associated with a reduced likelihood of the presence and severity of DR [24]. A sub-study of the PREDIMED randomized clinical trial showed that patients with type 2 diabetes who reported an intake of at least 500 mg/d of long-chain n3-PUFA at baseline had a 46% decreased risk of sight-threatening DR compared to those not meeting this target [25]. In an early stage of DR, supplementation with a high-dose DHA plus xanthophyll carotenoid multivitamin during 90 days was associated with a progressive and significant improvement of macular function measured by microperimetry [34]. In normal ocular tissues, angiogenic homeostasis is controlled by the balance between angiogenic stimulators, mainly VEGF, and angiogenic inhibitors such as pigment epithelium derived factor (PEDF) [49]. Moreover, this balance is important in the regulation of vascular permeability. While VEGF is increased in the vitreous of patients with diabetic macular edema, the vitreous level of PEDF is significantly lower in these patients [50]. Therefore, therapeutic strategies that can lower the VEGF/PEDF ratio are clinically beneficial. In a randomized single-blind controlled trial, the addition of a DHA dietary supplement to intravitreal ranibizumab (a monoclonal antibody against VEGF) was effective to achieve better sustained improvement of central subfield macular thickness outcomes after three years of follow-up compared with intravitreal ranibizumab alone [26]. Interestingly, n3-PUFA formulations in our study produced a small decrease in the VEGF/PEDF ratio, but the results were not statistically significant. Further studies analyzing the expression of VEGF and PEDF mRNA could help clarify the effect of EPA and DHA supplementation on the VEGF/PEDF ratio and their apparent clinical benefit in the treatment of diabetic macular edema.

As with any other clinical intervention, the safety of omega-3 dietary supplements should be considered. A recent meta-analysis that specifically addressed the safety and tolerability of prescription omega-3 fatty acids did not find any definitive evidence of serious adverse events [51]. The most commonly reported treatment associated adverse reactions are digestive disturbances (mainly dysgeusia or fishy taste) and skin reactions (eruption, itching, exanthema, or eczema). Although both EPA and DHA reduce the TG levels, they can increase the concentration of low density lipoproteins (LDL) [51]. However, this mild, but negative effect in lipid profile has not been observed in patients treated with supplements that only contain EPA. Monitoring of the lipid profile may be advisable in patients undergoing omega-3 supplementation.

To our knowledge, there has not been an in vitro study or clinical trial comparing the effects of so many different formulations of EPA and DHA on ARPE-19 cells, not only under normal conditions, but also following an oxidative challenge. It was very interesting to see that DHA and EPA had different effects when applied separately than they did when used in combination. Another relevant finding of our study was that the EE formulation had worse results, which cannot be explained by a lower bioavailability due to differences in digestion and absorption. This suggests that the EE form might decrease the biological effect of EPA and DHA. However, we know that our in vitro study has certain limitations. As an in vitro study, cells were not exposed to the same physiological environment as in the functioning retina. Moreover, analyzing the effect of the supplements in other retinal cell types would have also been of interest.

## 5. Conclusions

In summary, the present study demonstrates that different formulations of n3-PUFAs have a beneficial effect on cell viability and proliferation, and are able to restore oxidative induced RPE damage. The protective effects of these formulations on RPE cells may translate to effective treatments to prevent or delay DR progression, which will become increasingly important with the expected rise in DM prevalence and its associated socioeconomic burden in years to come. Although differences between the formulations in our study were small, our results are in accordance with previous reports suggesting that supplements that combine both n3-PUFAs with a higher proportion of EPA might be more beneficial, especially when present in the form of triglycerides or phospholipids. Clinical trials specifically designed to confirm these observations in patients with retinal vascular pathologies are needed.

## Figures and Tables

**Table 1:** Composition of the different groups of n3-PUFA (long-chain n-3 polyunsaturated fatty acids) supplements. All fatty acids (FA) are presented as a percentage of n3-PUFA, the remaining percentage in each group consisted of a non n3-PUFA diluent. EPA: Eicosapentaenoic acid; DHA: Docosahexaenoic acid; TG: Triglycerides; EE: Ethylesters; PL: Phospholipids.

	n3-PUFA Supplement ID	Composition
I	DHA	80% DHA (TG)
	EPA	80% EPA (TG)
II	EPA/DHA 40/20 TG	40% EPA: 20% DHA (TG)
	EPA/DHA 20/40 TG	20% EPA: 40% DHA (TG)
	EPA/DHA 40/20 EE	40% EPA: 20% DHA (EE)
III	DHA 97/3V	97% DHA (TG): 3% DHA (PL, vegetable)
	DHA 95/5V	95% DHA (TG): 5% DHA (PL, vegetable)
	DHA 97/3M	97% DHA (TG): 3% DHA (PL, marine)
	DHA 97/3 VM 1.5	97% DHA (TG): 3% DHA (PL, 1.5% vegetable + 1.5% marine)
	DHA 95/5 VM 2.5	95% DHA (TG): 5% DHA (PL, 2.5% vegetable + 1.5% marine)
